# Decoupling Fabrication From Encoding: DNA‐Addressable Template Microparticles for Large, User‐Defined Optical Barcode Libraries

**DOI:** 10.1002/advs.77407

**Published:** 2026-09-14

**Authors:** Akihiro Eguchi, Yuichiro Iwamoto, Hinata Tokuda, Haruka Narita, Adrian M. Martin, Sadao Ota

**Affiliations:** ^1^ Research Center for Advanced Science and Technology The University of Tokyo Tokyo Japan

**Keywords:** barcoding, DNA nanotechnology, high‐throughput screening, hydrogels, microfluidics

## Abstract

Optical barcodes for pooled high‐throughput screening must support large libraries while remaining decodable in a single imaging step. Existing approaches often trade design control for manufacturability: deterministic barcodes often require per‐code redesign of particle fabrication, whereas stochastic combinatorial barcodes are difficult to generate as predefined batches. Here we introduce a chemically programmable barcoding architecture that decouples particle fabrication from barcode assignment. Using a contact‐free multilaminar flow lithography platform with all‐around three‐dimensional sheathing, we continuously fabricate a universal hydrogel scaffold containing five spatially segregated DNA‐addressable domains at rates > 10^6^ particles/h. Chosen barcode identities are subsequently written on demand onto the same template batch by domain‐selective DNA hybridization. Single‐domain measurements resolved 64 candidate optical states, indicating an experimentally informed theoretical upper bound of 64^5^ ≈ 1.1 × 10^9^ barcodes. We further implemented a predefined 59,049‐code library by split‐pool labeling, achieving an 83.6% recovery of decoded beads at a stringent posterior threshold (≥ 0.95). After 11 days, >7,800 beads were correctly re‐identified at ≥ 0.95 accuracy in matched fields of view. This strategy provides a highly scalable, chemically programmable route to build large, user‐defined optical barcode libraries with single‐image optical readout and longitudinal traceability.

## Graphical abstract

A universal hydrogel microparticle template with five DNA‐addressable domains decouples particle fabrication from barcode writing. Contact‐free multilaminar flow lithography produces >106 templates/h, and post‐fabrication domain‐selective hybridization converts one batch into large, user‐defined optical barcode libraries with robust decoding and re‐identification.

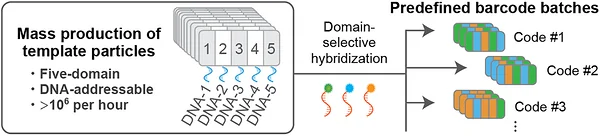

## 1 Introduction

High‐throughput screening increasingly relies on pooled workflows in which individual particles, reaction compartments, or payload carriers must be linked to machine‐readable identities that can be decoded rapidly in situ and revisited over time [1, 2, 3, 4, 5]. Optical barcodes are attractive for this purpose because they enable nondestructive readout from individual objects with traceability in the imaging field. In practice, however, the number of optical states that can be robustly distinguished from any single barcode feature is limited by spectral crosstalk, signal‐intensity variation, and measurement noise. Large optical barcode libraries that remain decodable in a single imaging step therefore require multiple independent barcode elements per particle, rather than assigning an increasing number of closely spaced states to a single optical feature.

Existing multielement barcoding strategies face a persistent trade‐off between design‐defined barcode identity and scalable library generation. In deterministic particle barcodes, code identity is defined during fabrication through particle geometry, prescribed spatial features, or fixed material composition [6, 7, 8, 9, 10, 11, 12, 13, 14, 15, 16, 17, 18, 19, 20, 21, 22]. While this approach provides designed, readily decodable barcodes, expanding the codebook requires redesign of fabrication patterns, flow configurations, or material states for additional codes. Conversely, stochastic approaches expand diversity through random combinatorial incorporation or mixing of barcode elements [23, 24, 25]. This approach can efficiently populate large code spaces, but the identity of each particle is set by stochastic assembly rather than being defined by design. Consequently, specific barcode species are difficult to generate on demand in defined quantities, complicating controlled library composition and one‐to‐one mapping between barcodes and chosen payloads.

What is needed, therefore, is a manufacturable particle architecture that combines the coding efficiency of multielement barcodes with the ability to specify barcode identity by design, as in deterministic single‐particle barcodes. We reasoned that this can be achieved if barcode identity is encoded by assigning one of N optical states to each of L ordered, independently addressable domains within a common template particle. This architecture expands coding capacity combinatorially, because assigning one of N states to each of L domains yields N^L^ distinguishable codes within a single‐particle format. It also enables direct preparation of defined batches of chosen codes from a common batch of identical templates, thereby decoupling particle fabrication from code assignment. Because the barcode is embedded in fixed domains of a single particle, the same architecture is naturally compatible with spatial fiducials, error‐aware decoding, and longitudinal re‐identification.

**FIGURE 1:**
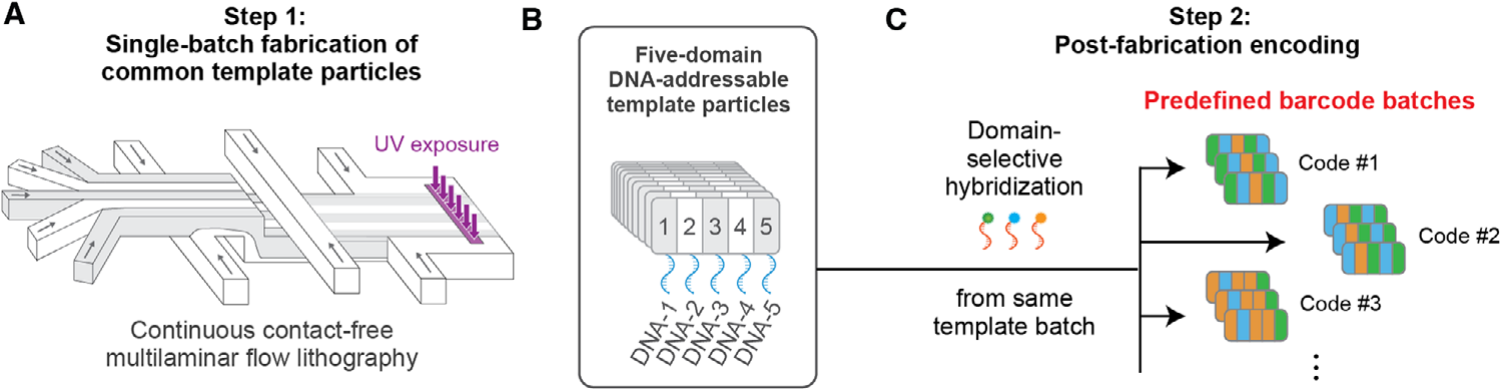
Large‐scale programmable optical barcoding by decoupling template‐particle fabrication from barcode assignment. (A) Single‐batch, high‐throughput fabrication of common hydrogel template particles containing five spatially segregated oligonucleotide domains by continuous contact‐free multilaminar flow lithography at rates of > 10^6^ particles/h. (B) The common template particles have five distinct layers, each functionalized with an orthogonal DNA sequence. (C) Post‐fabrication assignment of barcode identities by domain‐selective hybridization of fluorophore‐labeled complementary strands, enabling the same template batch to generate predefined barcode sets.

Here we implement this concept using hydrogel template microparticles that contain five spatially segregated DNA‐addressable domains (Figure 1). Each domain is defined by a distinct immobilized oligonucleotide handle, allowing barcode states to be written independently after particle fabrication by selective hybridization of fluorophore‐labeled complementary strands. Barcode identity is therefore not fixed during fabrication but assigned post‐fabrication across a universal particle scaffold. This architecture combines a common particle template with chemically programmable, domain‐resolved encoding.

A key challenge, however, is manufacturing such multidomain DNA‐addressable templates at scale while preserving domain‐level segregation. To address this challenge, we developed a contact‐free multilaminar flow lithography platform with all‐around three‐dimensional sheathing. The platform hydrodynamically isolates the photopolymerization zone from the channel walls and enables continuous fabrication of five‐domain particles with spatially segregated DNA‐addressable domains at > 10^6^ particles h^−1^. We then assigned barcode identities by domain‐selective hybridization, including predefined barcode batches from a common template and a 59,049‐code (9^5^) library generated by split‐pool labeling. Under a stringent posterior decoding threshold (≥ 0.95), 56,160 of 67,167 quality‐filtered beads (83.6%) were successfully decoded, and barcode identities remained sufficiently stable for reliable longitudinal re‐identification after 11 days. Together, these results establish a practical route to large, user‐defined optical barcode libraries that remain decodable in a single imaging step. The same template architecture also provides a chemically addressable foundation for future integration with functional payloads in screening workflows.

## 2 Results

### 2.1 High‐Throughput Fabrication of Five‐Domain DNA‐Addressable Template Microparticles via Contact‐Free Multilaminar Flow Lithography

The first experimental requirement for the barcoding architecture in Figure 1 is a scalable source of common template particles containing five independently addressable DNA domains. These particles must satisfy two conditions simultaneously: (i) they must preserve domain‐level segregation during fabrication so that each DNA domain can later be addressed independently, and (ii) they must be manufacturable at scale in a continuous, high‑throughput process compatible with large barcode libraries. This requirement is particularly stringent for miniaturized barcode particles. Smaller particles are advantageous for scalable barcoding because they can be produced in larger numbers, processed in smaller assay volumes, and imaged at higher particle densities. However, reducing particle dimensions also makes spatially encoded barcodes more sensitive to diffusion‐mediated blurring, because a given diffusion length represents a larger fraction of each encoded domain. Thus, scalable miniaturized barcodes require both high‐throughput particle production and preservation of sharply segregated DNA domains.

Flow lithography is a promising route to high‑throughput fabrication of spatially patterned functional hydrogel particles [26, 27, 28]. In particular, hydrodynamic focusing lithography (HFL) mitigates polymerization near the channel walls and the resulting risk of clogging by hydrodynamically separating the reactive stream from the device walls [29]. To date, multi‐domain HFL has predominantly been implemented in a stop‐flow configuration at a fabrication rate of 10^3^–10^4^/h [17, 30]. However, the overall monomer dwell times inherent to stop‑flow operation (typically around 500–1000 ms [17, 28, 30]) permit molecular diffusion across laminar interfaces. For oligonucleotide‐functionalized systems, this diffusion can broaden inter‐domain boundaries and compromise the domain‐level spatial segregation required for reliable post‐fabrication optical encoding. Moreover, we are not aware of reports demonstrating continuous HFL with more than three stacked laminar layers under continuous photopolymerization, highlighting the practical challenge of maintaining stable multilaminar streams in this regime.

**FIGURE 2:**
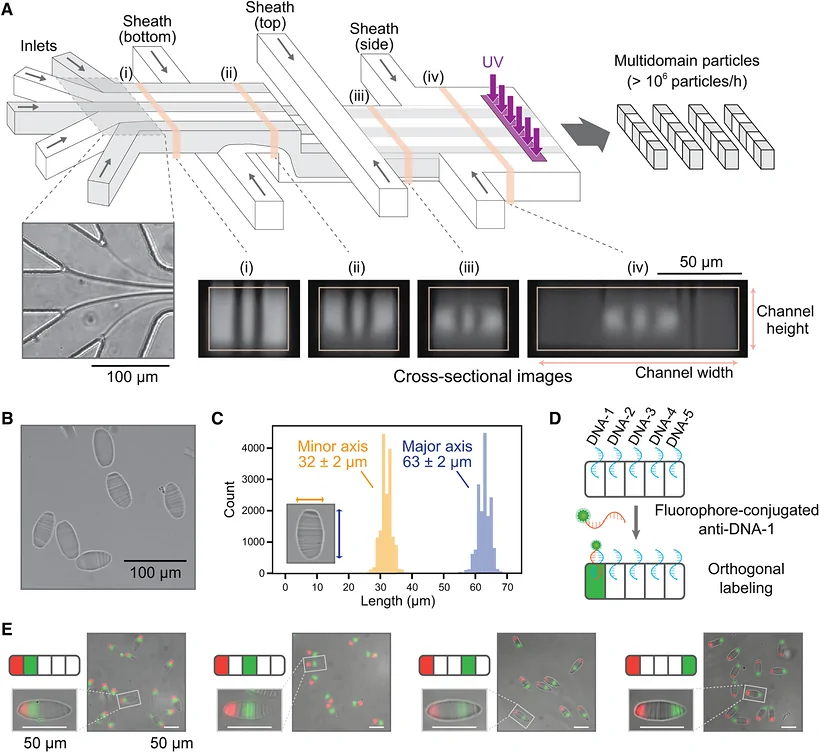
Contact‐free multilaminar flow lithography enables scalable fabrication of five‐domain DNA‐addressable template microparticles. (A) Two‐layer PDMS device design. Five reactive inlet streams form a stacked multilaminar core that is progressively surrounded by bottom, top, and lateral sheath flows (i–iv), generating a contact‐free reactive stream at the UV polymerization region; inset: bright‐field image of the multilaminar junction and representative cross‐sectional images along the channel. (B) Representative bright‐field image of fabricated five‐domain hydrogel template microparticles. (C) Size distributions of the major and minor particle axes measured from bright‐field images (*n* = 16,174). (D) Schematic of domain‐specific hybridization of fluorophore‐conjugated strands to particles bearing distinct immobilized anchor oligonucleotides. (E) Fluorescence/bright‐field overlays showing domain‐specific labeling of the intended domains after hybridization with fluorophore‐conjugated complementary strands; in each example, domain 1 was labeled with AF647 and one of domains 2–5 was labeled with AF488.

To address these constraints, we designed a relatively simple two‐layer bonded PDMS device that simultaneously generates five parallel reactive laminar streams and implements all‐around 3D sheathing of the reactive core (Figure 2A and Figure S1). The five streams define the five spatially segregated DNA‐addressable domains, while bottom, top, and lateral sheath flows progressively surround the multilaminar stream and hydrodynamically isolate it from all channel walls before UV exposure (bottom panels (i)‐(iv) of Figure 2A). This 3D sheathing architecture avoids deposition or adsorption of polymers on the channel walls and enables stable continuous operation. This geometry enables contact‐free continuous photopolymerization while maintaining the multilaminar organization at the polymerization region, thereby supporting stable high‐throughput fabrication of five‐domain template particles without requiring a complex three‐dimensional device assembly.

Using this device with spatially patterned strong 500‐µs UV pulses, we continuously generated five‐domain hydrogel microparticles at rates exceeding 10^6^ particles/h (Figure 2B). Stable operation was maintained for more than 3 h of continuous fabrication, yielding over 3 million beads in a single run. Bright‐field image analysis of the fabricated microparticles showed that the particles were highly uniform, with major and minor axes of 63 ± 2 and 32 ± 2 µm, respectively (Figure 2B,C), demonstrating that the reactive core stream can be stably maintained during continuous, long‐term fabrication. The short residence time before polymerization helps limit inter‐domain mixing. Under the flow conditions, the residence time in the lamination region was estimated to be approximately 42 ms (see Methods). Even using an upper‐bound diffusion coefficient for single‐stranded DNA in aqueous buffer (D ≈ 1.5 × 10^−10^ m^2^/s) [31], the estimated diffusion length over this interval is ∼3.5 µm, remaining below the 5 µm separation later used for domain segmentation in the decoding workflow, suggesting that diffusive broadening remained limited under the fabrication conditions used here. We next tested whether the embedded oligonucleotides remained chemically accessible and spatially resolved after polymerization. Hybridization of fluorophore‐labeled complementary strands produced fluorescence localized to the intended domains (Figure 2D,E), demonstrating domain‐resolved accessibility of the DNA anchors after continuous fabrication. Together, these results show that this platform can provide a scalable supply of uniform five‐domain template microparticles whose DNA‐defined domains remain spatially segregated and independently addressable for downstream post‐fabrication encoding.

To quantitatively evaluate the quality and reproducibility of the five‐domain templates, particles from three independent fabrication batches were stained in an alternating RGBRG pattern, enabling simultaneous visualization of all five domains and four inter‐domain boundaries (Figure S2). The width of each domain, quantified as the full width at half maximum (FWHM) of the fluorescence intensity profile, showed good particle‐to‐particle and batch‐to‐batch reproducibility, although the three internal domains were consistently wider than the two edge domains (Figure S2A). We further quantified fluorescence leakage between adjacent domains. For each adjacent pair, leakage from the source domain into the neighboring domain was calculated by dividing the source‐domain fluorescence measured at the center of the neighboring domain by that measured at the center of the source domain. Leakage was typically 10%–20% and was comparable across all adjacent domain pairs and fabrication batches (Figure S2B), supporting the reproducible formation of spatially resolved DNA‐addressable domains during continuous fabrication.

### 2.2 Programmable Post‐Fabrication Encoding and Per‐Domain Coding Capacity

**FIGURE 3:**
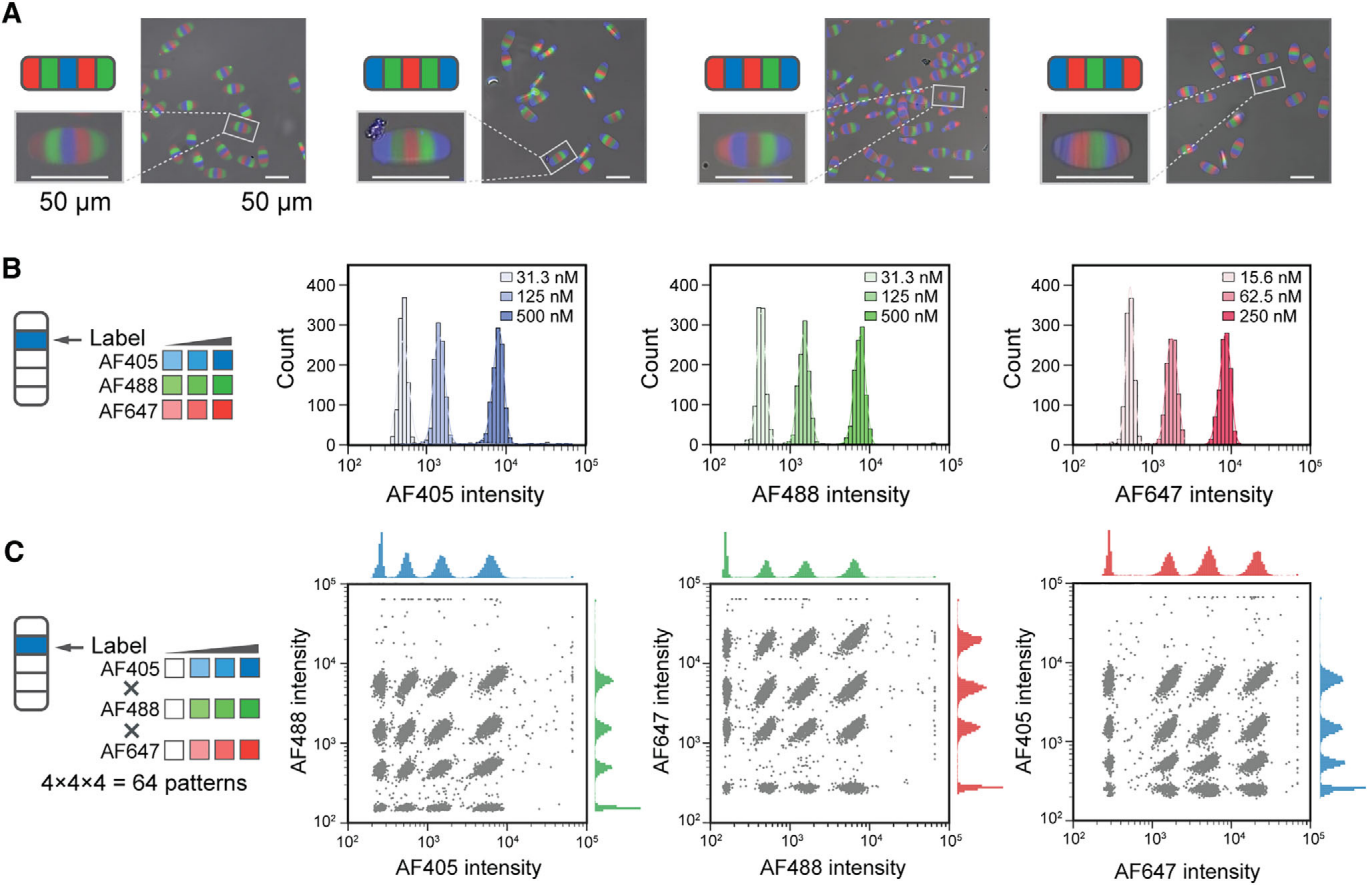
Programmable post‐fabrication encoding by domain‐selective hybridization. (A) Representative fluorescence overlays of distinct bead batches programmed to display predefined codes (blue: AF405, green: AF488, red: AF647). (B) Fluorescence intensity histograms showing three resolvable non‐zero levels per fluorophore obtained by titrating staining concentrations (values indicated), enabling four addressable levels including the unstained state (*n* = 1,000). (C) Pairwise intensity scatter plots for pooled 64 single‐domain states (4^3^ combinations of three fluorophores, each with four addressable levels), showing grid‐like cluster structure in AF405–AF488, AF488–AF647, and AF647–AF405 projections (*n* = 10,829).

Having established in Figure 2 that the template particles retain domain‐resolved DNA accessibility after continuous fabrication, we next asked whether these domains could be used to assign defined optical identities on a common particle template. To test this, a single batch of template particles was divided into four subsets and incubated with predefined cocktails of complementary oligonucleotides conjugated to the corresponding fluorophores: Alexa Fluor 405 (AF405), AF488, and AF647. This post‐fabrication labeling produced four distinct multidomain fluorescence patterns (Figure 3A). This result shows that predefined barcode batches can be generated on demand from the same fabricated particles solely by changing the labeling mixture, thereby decoupling particle fabrication from barcode assignment.

We then asked how many reliably distinguishable optical states could be assigned to an individual domain under standard fluorescence imaging. To estimate this per‐domain coding capacity, we tuned the concentrations of AF405‐, AF488‐, and AF647‐conjugated complementary oligonucleotides while maintaining the total oligonucleotide concentration constant. This produced three resolvable non‐zero intensity levels for each fluorophore (Figure 3B). Importantly, these intensity levels were reproducibly generated across three independent fabrication batches with low batch‐to‐batch variation (Figure S3). Together with the unlabeled state, this encoding scheme defines four addressable levels per fluorophore, and their combination yields a total of 4^3^ = 64 possible three‐color states per domain. When particles spanning this set were pooled, pairwise intensity plots showed the expected grid‐like cluster structure for each of the 64 fluorophore combinations (Figure 3C). Extrapolating the experimentally resolved 64 candidate states per domain across five independently addressable domains gives a theoretical upper estimate of 64^5^ ≈ 1.1 × 10^9^ barcodes. This value is an experimentally informed upper limit, whereas the operational library used below was restricted to a more conservative nine‐state‐per‐domain codebook to improve decoding robustness.

### 2.3 Decoding of a 59,049‐Code Library

We next asked whether the user‐defined encoding scheme remains reliably decodable at large library size. To test this, we processed template particles by a split‐pool workflow (Figure S4) with nine predefined per‐domain spectral states to generate a library with 9^5^ = 59,049 possible barcodes. We implemented the nine spectral states with nine distinct AF488 and AF647 labeling cocktails. Although the full theoretical design space consisted of 4^3^ = 64 states, we focused on the AF488 and AF647 channels for this proof‐of‐concept demonstration. Because the separation between the second and third fluorescence peaks was smaller than that between the first and second peaks, the third concentration level was omitted to reduce potential misclassification. Thus, three robust fluorescence levels were retained in each of the two channels, resulting in 3 × 3 = 9 states. AF405 was used exclusively as an internal spatial fiducial rather than as a coding channel: a stronger signal in domain 2 and a weaker signal in domain 4 provided a reference for domain ordering and segmentation.

**FIGURE 4:**
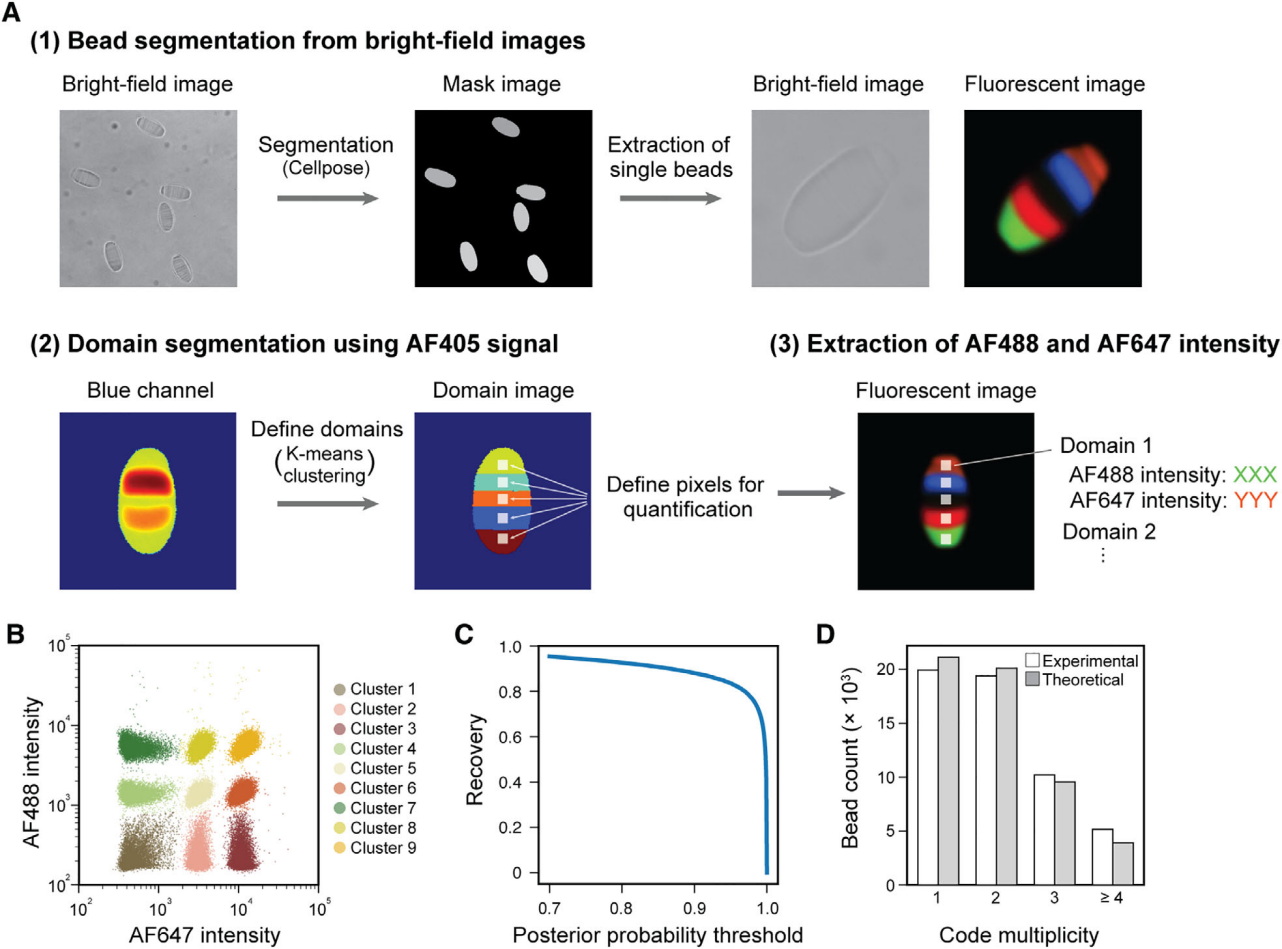
Decoding of a split‐pool library drawn from a predefined 59,049‐code codebook. (A) Image‐processing pipeline for domain‐resolved spectral intensity extraction: particle segmentation from bright‐field images, domain ordering and segmentation using AF405 fiducial domains, and quantification of AF488 and AF647 intensities from each of the five domains. (B) Representative AF647–AF488 intensity scatter plot for a single domain, showing nine spectral clusters assigned by a Gaussian mixture model (GMM). (C) Decoding recovery as a function of the posterior assignment probability threshold, with full‐particle calls retained only when all five domains exceed the threshold. (D) Distribution of observed barcode multiplicity, defined as the number of beads assigned to the same decoded code, compared with the Poisson expectation for uniform random sampling from a 59,049‐code codebook.

For decoding, individual particles were first identified in bright‐field images, matched to the corresponding fluorescence images, and subjected to quality filtering (see Methods). To quantify the practical throughput of the workflow, we tracked particle numbers across the fabrication, split‐pool encoding, imaging, quality filtering, and final decoding steps. In this experiment, 1,584,000 particles were fabricated, 180,000 particles were used for split‐pool encoding, and 145,000 particles were recovered after encoding. Of these, 79,342 particles were detected in the acquired images based on Cellpose segmentation; 67,167 particles exhibited the correct five‐domain morphology, passed quality filtering, and were used for fluorescence intensity extraction (Table S1). The five domains were then segmented based on the AF405 fiducial signals, and the AF488 and AF647 intensities of each domain were extracted for 67,167 beads (Figure 4A).

For each domain, we modeled the AF488 and AF647 intensity distribution using a Gaussian mixture model (GMM) with nine components corresponding to the nine spectral states (Figure 4B and Figure S5). We then computed the posterior probabilities for each state assignment and used the maximum posterior probability as a measure of assignment confidence. Full‐particle calls were retained only when all five domains exceeded a posterior threshold of 0.95. Under this stringent criterion, 56,160 out of 67,167 quality‐filtered beads were successfully decoded, corresponding to an 83.6% recovery (Figure 4C). When the other 16.4% low‐confidence beads were highlighted on the AF488–AF647 scatter plots (Figure S6), they were enriched near inter‐cluster boundaries. Thus, the posterior threshold mainly removed beads with ambiguous spectral‐state assignments, rather than selectively excluding dim or poorly imaged particles.

To examine whether the split‐pool process generated the 59,049‐code library without strong representation bias, we first quantified the actual observed barcode diversity among high‐confidence decoded beads. Of the 56,160 beads passing the posterior threshold, 35,155 unique barcode identities were observed, corresponding to 59.5% coverage of the designed 59,049‐code space and a missing‐code fraction of 40.5%. Under a simple uniform occupancy model, the expected number of unique barcode identities was 36,237; the experimentally observed value was approximately 3.0% lower than this expectation. We then examined barcode multiplicity, defined as the number of beads assigned to the same decoded code. The observed multiplicity distribution closely followed the Poisson expectation for uniform random sampling from a 59,049‐member library (Figure 4D). This agreement suggests that the sampled population was broadly consistent with approximately uniform sampling from the predefined codebook without evident strong representation bias.

### 2.4 Longitudinal Re‐Identification After 11 Days of Storage

**FIGURE 5:**
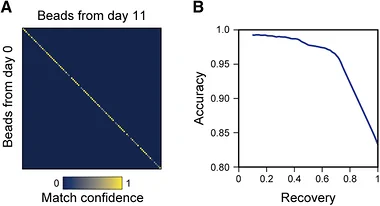
Longitudinal re‐identification after 11 days of storage. (A) Heatmap of pairwise match‐confidence matrix between the initial and 11‐day datasets, shown for a 100‐pair subset of the positional ground‐truth set (10,654 matched bead pairs) and ordered according to the true bead correspondence. The strong diagonal indicates correct re‐identification. (B) Accuracy–recovery curve obtained by thresholding the match‐confidence score, wherein accuracy is defined as the fraction of retained matches consistent with the positional ground truth, and recovery as the fraction of the 10,654 matched bead pairs retained above threshold.

For pooled screening applications, barcode readout must remain stable enough to support re‐identification after long imaging intervals. We therefore imaged an independently prepared library before and after 11 days of storage at 4°C in TET‐SC buffer in the dark and constructed a positional ground‐truth set of 10,654 matched bead pairs by identifying beads that remained at the same field positions in both imaging sessions. The fluorescence intensity distributions of AF488 and AF647 showed substantial overlap between days 0 and 11 (Figure S7). We then performed probabilistic matching using the full code‐assignment probability vectors obtained from the GMM/Bayesian inference framework, allowing a match‐confidence score to be computed for each candidate pair. When the pairwise match‐confidence matrix was ordered according to the positional ground truth, the resulting heatmap showed a strong diagonal correspondence (Figure 5A and Figure S8), indicating that barcode readout remained sufficiently stable to discriminate correct bead identities after storage. Varying the match‐certainty threshold allowed us to quantify the trade‐off between re‐identification accuracy and recovery (Figure 5B). At thresholds that maintained accuracy above 0.95, more than 7,800 beads were correctly re‐identified, corresponding to over 73% of a positional ground‐truth set of 10,654 matched bead‐pairs. These results show that the DNA‐linked spectral barcode readout remains sufficiently stable to support high‐confidence longitudinal re‐identification over at least 11 days, while ambiguous cases can be excluded by probabilistic confidence filtering.

## 3 Discussion

We developed a barcoding architecture in which five‐domain DNA‐addressable hydrogel template microparticles are fabricated continuously at high throughput as a common template, and optical identities are assigned afterward by domain‐selective hybridization. Using contact‐free multilaminar flow lithography with all‐around three‐dimensional sheathing, we continuously fabricated template particles at > 10^6^ particles/h while preserving domain‐level DNA addressability. On this scaffold, we demonstrated programmable post‐fabrication encoding, established an experimentally informed upper‐bound capacity of 64^5^ ≈ 1.1 × 10^9^ possible barcodes based on the resolved single‐domain state space, and implemented a conservative 59,049‐code (9^5^) library optimized for robust decoding. At a stringent posterior threshold, 83.6% of quality‐filtered beads were successfully decoded, and more than 7,800 beads were correctly re‐identified after 11 days at ≥ 0.95 accuracy.

To better position this work relative to prior microfluidic optical barcode particle technologies, we compared representative approaches in terms of encoding principle, whether a batch carrying a preselected code can be generated directly, the number of distinct codes obtainable from a single particle‐fabrication condition, particle‐fabrication throughput, and particle dimensions (Table 1). Graphically encoded hydrogel particles and microfluidically produced spectral beads can generate batches carrying predefined optical codes; however, each fabrication or synthesis condition generally produces only one code because the optical identity is established during particle formation through a code‐specific photomask, exposure pattern, flow configuration, or material composition. By contrast, stochastic combinatorial systems can generate many codes in a single fabrication process, but the code assigned to each particle is probabilistic, and a batch carrying a preselected code cannot generally be produced directly. The principal conceptual advance of the present platform is the separation of common‐template fabrication from final optical code assignment. A common population of five‐domain DNA‐addressable template particles is first continuously produced under a single fabrication condition, after which optical identities are assigned by domain‐selective DNA hybridization. The same template population can therefore be used in two complementary modes: direct labeling to generate batches carrying preselected optical codes and split–pool labeling to construct a combinatorial library sampled from a predefined space of 59,049 possible codes. Consequently, the number of particle‐fabrication conditions does not need to increase one‐to‐one with the number of final optical codes. This post‐fabrication encoding strategy is enabled by an all‐around three‐dimensional sheathing architecture that permits continuous production of the five‐domain templates at approximately 10^6 particles/h, with particle dimensions of approximately 63 × 32 µm.

**TABLE 1:** Comparison of representative microfluidic optical barcode particle technologies: Graphically encoded hydrogel particles, including systems reported by Doyle and co‐workers, microfluidically produced spectral beads, stochastic combinatorial optical barcodes, and our work. The table compares representative approaches in terms of whether each can generate a batch carrying a preselected code, barcode diversity obtainable from one particle‐fabrication condition, particle fabrication throughput, and particle size and shape. *(n) is the number of available barcode components and (m) is the number incorporated into each particle. ^†^A preselected‐code batch can be prepared by direct post‐fabrication labeling. In the split‐pool mode, individual bead codes are assigned combinatorially. ^‡^(N) is the number of post‐fabrication states per domain and (L) is the number of independently addressable domains. The value 35,155 is the number of unique identities observed among 56,160 high‐confidence decoded beads sampled from the predefined 59,049‐code codebook.

Approach (representative refs.)	Can a batch carrying a preselected code be prepared?	Barcode diversity obtainable from one particle‐fabrication condition	Particle fabrication throughput	Particle size and shape
Graphically encoded hydrogel microparticles by flow lithography [10, 12, 13, 16, 17]	Yes	1	∼ 1 × 10^4^ /h	250–400 µm × 70–100 µm plate‐like
Microfluidic spectral beads such as MRBLE [20, 21]	Yes	1	∼ 9 × 10^6^ /h	50 µm diameter sphere
Stochastic combinatorial optical barcodes [24]	No	C(n,m)*	∼ 1 × 10^6^ /h	75 µm diameter sphere
This work	Yes^†^	N^L^: 9^5^ = 59,049 codebook; 35,155 unique identities observed^‡^	∼ 1 × 10^6^ /h	63 µm × 32 µm plate‐like

Decoupling barcode assignment from fabrication does not remove manufacturing demands; it relocates them. Library performance still depends on uniform particle geometry, preserved domain segregation, accessible chemical addresses, and low defect rates. More broadly, it also differs fundamentally from sequencing‐based strategies such as DNA‐encoded libraries, in which identity is recovered by destructive sequencing. Here, DNA serves instead as a programmable chemical address for optical signals, enabling direct, in situ readout and longitudinal traceability.

Finally, the present implementation should be viewed as a modular foundation rather than an endpoint specific to DNA hybridization. Although barcode assignment here was performed by complementary oligonucleotide labeling, the same decoupled template architecture should in principle accommodate alternative post‐fabrication chemistries, including covalent strategies compatible with harsh reaction conditions. More generally, the ordered address domains provide a natural basis for future coupling of barcode identity to functional payloads, offering a route toward user‐defined code–payload mappings in pooled screening, phenotypic selection, and spatially resolved assay formats.

## 4 Experimental Section

### 4.1 Materials

All oligonucleotide reagents (see also Tables S2 and S3) were purchased from Integrated DNA Technologies (IDT) or Eurofins. Poly(ethylene glycol) diacrylate (Mn = 575; PEGDA575) was obtained from Sigma–Aldrich. Poly(ethylene glycol) (PEG200) was purchased from FUJIFILM Wako Pure Chemical Corporation. 2‐Hydroxy‐2‐methylpropiophenone (HMPP) was obtained from Tokyo Chemical Industry (TCI). A blocking reagent CELLOTION was purchased from Takara Bio Inc.

### 4.2 Microfluidic Device Fabrication

The microfluidic devices were fabricated in polydimethylsiloxane (PDMS; SILPOT 184, Dow Corning) using standard soft lithography. Two device layers were fabricated separately and manually aligned before bonding. The device has five sample inlets for the PEGDA solution, three sheath inlets, and one outlet. The five input samples join together first, followed by sequential confluence with bottom, top, and side sheath solutions. All three sheath streams were split into two branches immediately after inlet entry and merged with the sample stream from both sides.

### 4.3 Theoretical Estimation of Inter‐Domain Diffusion

To assess the potential for diffusive mixing between adjacent laminar flows prior to polymerization, we estimated the diffusion length (*L_d_*) using the Einstein‐Smoluchowski relation: *L_d_* =$\sqrt{2Dt}$, where *D* is the diffusion coefficient and *t* is the residence time. Based on the experimental flow rate (*Q* = 1.0 µL/min) and the cross‐sectional area of the focused sample stream (*A* ∼ 50 × 20 µm^2^), the flow velocity *v* is calculated as:

$$v=\frac{Q}{A}\;\;∼\;1.67\;\times \;10^{-2}\;m/s$$

The distance from the confluence point to the UV irradiation zone is *d* = 700 µm. Thus, the residence time *t* is:

$$t=\frac{d}{v}\;∼\;0.042\;s$$

Using the diffusion coefficient of a typical short oligonucleotide (D ≈ 1.52× 10^−10^ m^2^/s), the diffusion length is calculated as:

$$\;L_{d}=\sqrt{2\;\times \;1.52\times 10^{-10}\times 0.042}\;\;∼\;3.5\;\mu m$$

*Note*: We used the diffusion coefficient of DNA in a standard aqueous buffer. Since the viscosity of the 40% PEGDA solution is higher than that of the buffer, the actual diffusion coefficient in the microfluidic channel is likely lower, making this a conservative estimate of the broadening.

### 4.4 Particle Generation

Used for Figures 2 and 5. All the solutions were sent to the device through PEEK tubes. The 40% PEGDA575 solutions with 5% HMPP and specific capture oligonucleotides (see Table S2) in DPBS were introduced to the device using a FLPG Plus (Fluigent, FLPG005J) and a LineUp Flow EZ (Fluigent, LU‐FEZ‐2000) at 6–15 mbar. To achieve contact‐free 3D focusing, sheath solutions, 20% PEG200 in DPBS, are sent from syringes to the device using syringe pumps (Harvard Apparatus, PUMP 11 ELITE). The flow rates are 0.8–1.2 µL/min for the bottom and top sheath, and 2.4–3.0 µL/min for the side sheath. The laser beam (375 nm) to induce polymerization of the PEGDA solution was shaped into a rectangular profile of approximately 150 µm × 10 µm using a cylindrical lens and a spatial filter, and then tightly focused onto the channel by a high‐NA (0.8) objective lens, enabling pulsed UV irradiation at 100–1000 Hz synchronized with the sample flow. The particles were washed with and kept in the TET buffer (20 mM Tris‐HCl (pH 8.0), 0.5 mM EDTA, and 0.01% Tween 20) or TET‐SC buffer (20 mM Tris‐HCl (pH 8.0), 50 mM NaCl, 0.5 mM EDTA, 0.01% Tween 20, and 50% Cellotion).

### 4.5 Particle Generation

Used for Figures 3 and 4 and Figure S2 and S3. All the solutions were sent to the device through PEEK tubes. To achieve contact‐free 3D focusing, the 40% PEGDA575 solutions with 5% HMPP and specific capture oligonucleotides (see Table S3) in DPBS and sheath solutions (10% PEG200 in DPBS) are sent from syringes to the device using syringe pumps (Harvard Apparatus, PUMP 11 ELITE). The flow rates are 0.4 µL/min for the samples, 3 µL/min for the bottom and top sheath, and 6–10 µL/min for the side sheath. The laser beam (375 nm) to induce polymerization of the PEGDA solution was shaped into a rectangular profile of approximately 150 µm × 10 µm using a cylindrical lens and a spatial filter, and then tightly focused onto the channel by a high‐NA (0.8) objective lens, enabling pulsed UV irradiation at 300 Hz synchronized with the sample flow. The particles were washed with and kept in the TET‐SC buffer (20 mM Tris‐HCl (pH 8.0), 50 mM NaCl, 0.5 mM EDTA, 0.01% Tween 20, and 50% Cellotion).

### 4.6 Domain‐Specific Particle Coloring

The beads were incubated for 3 h in TET‐SC buffer with 2 µM AF647‐conjugated anti‐DNA‐1 and 2 µM of either AF488‐conjugated anti‐DNA‐2, anti‐DNA‐3, anti‐DNA‐4, or anti‐DNA‐5 (Figure 2). The incubation was performed at ambient temperature in the dark. After incubation, the beads were washed three times with TET‐SC buffer (centrifuge: 100 × g, 3 min) and resuspended in the same buffer before imaging. The images were acquired using an INCell Analyzer 6000.

### 4.7 Quantification of Domain Width and Inter‐Domain Fluorescence Leakage

The beads were incubated for 3 h in TET‐SC buffer with 1 µM of AF647‐conjugated anti‐DNA‐1, AF488‐conjugated anti‐DNA‐2, AF405‐conjugated anti‐DNA‐3, AF647‐conjugated anti‐DNA‐4, and AF488‐conjugated anti‐DNA‐5 (Figure S2). The incubation was performed at ambient temperature in the dark. After incubation, the beads were washed three times with TET‐SC buffer (centrifuge: 100 × g, 3 min) and resuspended in the same buffer before imaging. The images were acquired using an INCell Analyzer 6000. Fluorescence intensity profiles were extracted from rotationally aligned particle images along the longitudinal axis of each particle. At each longitudinal position, fluorescence intensity was averaged over a five‐pixel‐wide band centered on the particle centerline. Intensity profiles were obtained separately for the red, green, and blue fluorescence channels.

Because the particles were stained in an alternating RGBRG pattern, the red and green intensity profiles were each fitted with two Gaussian functions, whereas the blue intensity profile was fitted with one Gaussian function. Each fluorescence profile was fitted by nonlinear least‐squares regression using a sum of Gaussian functions and a constant baseline:

$$I_{c}\left(x\right)=b_{c}+\sum_{k}A_{c,k}exp\lceil -\frac{{\left(x-\mu_{c,k}\right)}^{2}}{2\sigma_{c,k}^{2}}\rceil$$

where *I_c_* (x) is the fluorescence intensity in channel *c*, A*_c,k_*, *µ_c,k_*, and *σ_c,k_* are the amplitude, center position, and standard deviation of Gaussian peak *k*, respectively, and *b_c_* is the baseline offset. Particles were excluded when the detected number of peaks differed from that expected for the corresponding fluorescence channel or when the coefficient of determination of the fit was below *R^2^* = 0.975.

The five fitted peaks were assigned to domains 1–5 according to their spatial order and the RGBRG staining pattern. Particles were retained only when all five domains were identified in the expected spatial order, either domains 1–5 or domains 5–1 depending on particle orientation. Particles containing missing fit parameters were excluded. To remove particles with atypical segmentation, particles with mask areas outside the mean ± 2 standard deviations of the analyzed population were also excluded. Domain width was defined as the full width at half maximum of the corresponding fitted Gaussian:

$$FWHM_{i}=2\surd 2ln2\sigma_{i}$$

For each pair of adjacent domains, fluorescence leakage from source domain *i* into neighboring domain *j* was quantified from the fitted Gaussian for the source domain. Leakage was defined as the fitted source‐domain fluorescence at the center position of the neighboring domain, normalized to the fitted peak amplitude of the source domain:

$$L_{i\to j}\left(\%\right)=100\times \frac{G_{i}\left(\mu_{j}\right)}{A_{i}}$$

where *G_i_(x)* is the Gaussian fitted to source domain *i*, *A_i_* is its fitted peak amplitude, and *µ_j_* is the fitted center position of neighboring domain *j*. Leakage was calculated in both directions across each of the four adjacent‐domain boundaries. The distributions of domain widths and directional leakage values were visualized as violin plots for three independent fabrication batches.

### 4.8 Concentration‐Tuned Encoding

The beads were incubated overnight in TET‐SC buffer with a 1.5 µM oligonucleotide cocktail consisting of AF647‐conjugated (0, 15.6, 62.5, or 250 nM), AF488‐conjugated (0, 31.3, 125, or 500 nM), AF405‐conjugated (0, 31.3, 125, or 500 nM), and non‐labeled anti‐DNA‐2 oligonucleotides (Figure 3). Non‐labeled oligonucleotides were added so that the total oligonucleotide concentration remained constant across all staining conditions. The incubation was performed at ambient temperature in the dark. After incubation, the beads were washed three times with TET‐SC buffer (centrifuge: 100 × g, 3 min), pooled, and resuspended in the same buffer before imaging. The images were acquired using an INCell Analyzer 6000 with autofocus based on the blue channel detecting the AF405 signal.

### 4.9 Split‐Pool Combinatorial Encoding

A library of encoded beads was generated via a split‐pool method targeting 9 distinguishable states per domain (Figure 4). Initially, 1.8 × 10^5^ beads were divided into nine wells and incubated for 3 h with an oligonucleotide cocktail containing distinct ratios of AF647‐, AF488‐, AF405‐, and non‐labeled oligonucleotides in TET‐SC buffer. The non‐labeled oligonucleotides were supplemented to maintain a constant total oligonucleotide concentration across all staining conditions. After incubation, the beads were washed three times with TET‐SC buffer (centrifuge: 100 × g, 3 min), pooled into a single reservoir, and re‐aliquoted into nine wells for the subsequent layer encoding. This cycle was repeated for all designated domains, and details of the staining conditions are provided in Table S4.

The images were acquired using an INCell Analyzer 6000 with autofocus based on the blue channel detecting the AF405 signal. We corrected chromatic aberration between the three channels with standard white agarose beads by applying an affine transformation using pyStackReg (v0.2.8), aligning the AF405 and AF647 channels to the AF488 channel. To exclude defocused images, we developed a three‐layer CNN classifier trained on 52 and validated on 14 human‐annotated bright‐field images (35 focused and 31 defocused in total). Each input was a 256 × 256 pixel, intensity‐normalized patch cropped from 2,048 × 2,048 pixel images divided into 8 × 8 patches. For each acquired image, the classifier predicted focus labels for all 64 patches, and the overall focus state was determined by majority voting. We used 267 images that were classified as in focus of the 289 images acquired.

### 4.10 Bead and Domain Segmentation for Decoding Color‐Code

The following image analyses were performed in Python 3.13 (Figure 4). The analysis environment and all scripts are available at https://github.com/solabtokyo‐org/PEGDABar‐analysis.

Beads were segmented from focused bright‐field images using Cellpose (v4.2.1) [32] with the following parameters: flow_threshold = 0.4, cellprob_threshold = 0.0, tile_norm_blocksize = 0, and batch_size = 32. For each segmented bead, a bounding box was defined from the mask coordinates and expanded by 48 pixels on each side. The cropped image was then aligned by ellipse fitting and rotation so that the major axis became vertical.

Domain segmentation within each bead was performed using its bead mask and AF405 channel image. Prior to segmentation, bright fluorescent debris was identified within the bead mask using an AF405 intensity threshold of 12,000, which was determined from the distribution of the maximum AF405 intensity of domain 2 across multiple fields of view (approximately the mean + 3 SD; 11,871). The detected debris (pixel intensity > 12,000) was dilated by four iterations and replaced with the median intensity of the remaining pixels to prevent artifactual clustering. AF405‐labeled domains—candidates for domains 2 and 4—were then segmented in two steps. First, pixels were grouped into intensity‐based clusters (k‐means, *n* = 5) using log‐transformed AF405 intensity; the cluster with the highest mean intensity was decomposed into connected components, and the component without internal holes was assigned as domain 2. Second, the remaining unassigned pixels were grouped into intensity‐based clusters (k‐means, *n* = 3) using linear‐scale AF405 intensity. Morphological features (area, number of holes, circularity) were calculated for each cluster, and those with area ≥ 50 pixels, no internal holes, and circularity ≥ 0.5 were retained as candidates for domain 4; among these, the component with the highest mean AF405 intensity was assigned as domain 4. Each selected domain was horizontally expanded to cover the full bead width based on its vertical coordinate range, yielding five domains per bead. For the remaining three domains, pixels with AF405 intensities greater than the mean + 15 σ of the background—calculated from regions outside the bead mask—were excluded from these candidate domains.

Before color‐code assignment, we corrected fluorescence crosstalk between adjacent domains and intensity variation associated with domain width. All correction steps were performed in log‐intensity space.

Crosstalk was estimated from the geometry of the joint intensity distributions of neighboring domains. Density modes defining the intensity grids were detected by two‐dimensional kernel density estimation (KDE) for domains 1–2 and 4–5 and by three‐dimensional KDE for domains 2–4. Along each coordinate axis, the detected modes were ranked and divided into three intensity levels.

For each domain, the corresponding grid‐line vectors were averaged and normalized to 1 in that domain, with the remaining components defining the crosstalk coefficients. Coefficients for domains 1–2 and 4–5 were estimated first. Their estimated contributions were then removed from domains 2 and 4 before estimating the coefficients among domains 2–4. The five normalized direction vectors formed a 5 × 5 mixing matrix.

Domain width was normalized within each domain by its median and then natural‐log transformed. For each fluorescence channel and domain, width‐associated intensity variation was estimated by ordinary least‐squares regression with the KDE‐derived five‐domain intensity‐population combinations as fixed effects. The fitted width‐dependent component was subtracted from the observed log intensity.

Population assignments, crosstalk coefficients, and width coefficients were iteratively re‐estimated. Convergence was reached when the overall proportion of bead–domain assignments that changed between successive iterations was no greater than 0.1% and the maximum absolute changes in the crosstalk and width coefficients were no greater than 10^−4. A maximum of 20 iterations was used.

Finally, the width‐adjusted log intensities were centered by subtracting the per‐domain medians of the width‐adjusted training data. Crosstalk‐corrected intensities were then obtained by solving the linear system defined by the final mixing matrix.

### 4.11 Color‐Code Assignment

For each domain of each bead, the integrated intensities of the AF405, AF488, and AF647 channels were calculated within a 5‐pixel window centered at the domain centroid (Figure 4). Domain order was determined according to AF405 intensity (domain 2> domain 4> others).

We then employed a Gaussian mixture model (GMM) to assign AF488/AF647‐defined color codes. The parameters of the GMM, with the number of expected color codes set as the number of components, were estimated from log_10_‐transformed AF488 and AF647 intensities using the expectation–maximization algorithm as implemented in scikit‐learn (v1.7.1). The fitted GMM defines, for each color code *c*, the likelihood p(x*_i,l_* |*c*) of observing the intensity vector x*_i,l_* for bead *i* and domain *l*. Using the mixture weights as priors, we computed the posterior probability that domain *l* of bead *i* belongs to code *c* denoted as D_1_[*i*,*l*,*c*].

For each bead *i* and domain *l*, the color code with the highest posterior probability was assigned if this value exceeded a predefined threshold; otherwise, the bead was excluded as an outlier. Consequently, each bead was represented by a set of five domain‐level color codes with associated posterior probabilities, forming its barcode identity.

### 4.12 Bead Matching

A library was generated according to the “Split‐pool combinatorial encoding” section, and details of the staining conditions are provided in Table S5. For the analysis of bead matching, the beads were imaged before storage, stored at 4°C in TET‐SC buffer in the dark for 11 days, and then re‐imaged for the analysis shown in Figure 5. After the image acquisition, we used 628 images that were classified as in focus of the 800 images acquired.

Bead segmentation and domain segmentation were done following the “Bead and domain segmentation for decoding color‐code” protocol with slight modification. Specifically, the bead segmentation was done by using Cellpose version 4.0.6. AF405‐labeled domains—candidates for domains 2 and 4—were segmented by applying k‐means clustering (*n* = 6) to the logarithmically transformed AF405 channel image, and morphology‐based filtering was done with criteria of ≥ 100 pixels and circularity of 0.3–0.9.

For color code assignment, outlier beads were excluded based on histogram‐derived intensity gates: beads with domain 2 AF405 intensity between 4,175 and 26,053 and domain 4 intensity between 800 and 2,000 were retained.

To identify corresponding beads between images independently acquired on D1 and D2, we applied a matching algorithm based on joint‐color‐code assignment likelihood between every pair of bead *i* from D1 and bead *j* from D2. For each acquisition n, the posterior probability that the *k*‐th domain of bead *i* has color code *c* was denoted as *p* *_n, ik_* (*c*). The matching likelihood between bead *i* and bead j was defined in the log domain as P(*i*, *j*) = Σ*l* log(D1[*i*, *l*,:] D2[*j*, *l*,:]^T), representing the marginal log‐likelihood of domain‐wise color‐code consistency between the two beads.

$$P\;\left(i,j\right)=\sum_{k=1}^{5}\log\left(\;\sum_{c=1}^{9}p_{1,\mathrm{ik}}\left(c\right)p_{2,\mathrm{jk}}\left(c\right)\right)$$

For each bead *i*, the values P(*i*,*j*) were normalized across all *j* using the softmax function so that ∑*j*P(*i*,*j*) = 1 and the resulting softmax‐normalized values were used as matching certainties.

### 4.13 AI Usage

The authors used ChatGPT Pro (OpenAI) for English language editing. All content was reviewed and verified by the authors.

### 4.14 Statistical Analysis

Data preprocessing, including image registration, intensity transformation and normalization, fluorescence correction, and exclusion based on predefined quality criteria, was performed as described in the corresponding subsections.

Unless otherwise stated, n denotes the number of individual particles analyzed, whereas the number of independent fabrication batches is reported separately.

No formal hypothesis testing was performed; therefore, p values and significance thresholds were not applicable. Analyses were performed in Python 3.13 using custom scripts, pyStackReg (v0.2.8), Cellpose (v4.2.1 or v4.0.6), and scikit‐learn (v1.7.1).

## Author Contributions

**Haruka Narita**: methodology, software, writing – review and editing, investigation. **Adrian M. Martin**: methodology, investigation. **Sadao Ota**: conceptualization, writing – original draft, project administration, supervision, visualization, writing – review and editing, funding acquisition. **Hinata Tokuda**: methodology, software, investigation, writing – review and editing. **Akihiro Eguchi**: conceptualization, methodology, funding acquisition, writing – original draft, visualization, investigation. **Yuichiro Iwamoto**: writing – review and editing, software, investigation, methodology.

## Conflicts of Interest

The authors declare no conflicts of interest.

## Supporting information




**Supporting File**: advs77407‐sup‐0001‐SuppMat.docx.

## Data Availability

The data that support the findings of this study are available from the corresponding author upon reasonable request.

## References

[1] R. J. Fulton , R. L. McDade , P. L. Smith , L. J. Kienker , and J. R. Kettman Jr , “Advanced Multiplexed Analysis With the FlowMetrix^TM^ System,” Clinical Chemistry 43, no. 9 (1997): 1749–1756, 10.1093/clinchem/43.9.1749. 9299971

[2] S. C. Chapin and P. S. Doyle , “Ultrasensitive Multiplexed microRNA Quantification on Encoded Gel Microparticles Using Rolling Circle Amplification,” Analytical Chemistry 83, no. 18 (2011): 7179–7185, 10.1021/ac201618k. 21812442 PMC3173568

[3] B. Houser , “Bio‐Rad's Bio‐Plex Suspension Array System, xMAP Technology Overview,” Archives of Physiology and Biochemistry 118, no. 4 (2012): 192–196, 10.3109/13813455.2012.705301. 22852821 PMC3469222

[4] H. J. Moon , S. J. Mun , J. H. Lee , Y. H. Roh , Y. J. Lim , and K. W. Bong , “Encoded Hydrogel Microparticles With Universal Mismatch‐incorporated DNA Probes for Highly Specific Multiplex Detection of SNPs,” Talanta 245 (2022): 123480, 10.1016/j.talanta.2022.123480. 35462139

[5] J. B. Hein , H. T. Nguyen , D. H. Garvanska , et al., “Phosphatase Specificity Principles Uncovered by MRBLE:Dephos and Global Substrate Identification,” Molecular Systems Biology 19, no. 12 (2023): 11782, 10.15252/msb.202311782. PMC10698503 37916966

[6] M. Han , X. Gao , J. Z. Su , and S. Nie , “Quantum‐dot‐tagged Microbeads for Multiplexed Optical Coding of Biomolecules,” Nature Biotechnology 19, no. 7 (2001): 631–635, 10.1038/90228. 11433273

[7] S. R. Nicewarner‐Pena , R. G. Freeman , B. D. Reiss , et al., “Submicrometer Metallic Barcodes,” Science 294, no. 5540 (2001): 137–141, 10.1126/science.294.5540.137. 11588257

[8] F. Cunin , T. A. Schmedake , J. R. Link , et al., “Biomolecular Screening With Encoded Porous‐silicon Photonic Crystals,” Nature Materials 1, no. 1 (2002): 39–41, 10.1038/nmat702. 12618846

[9] M. J. Dejneka , A. Streltsov , S. Pal , et al., “Rare Earth‐doped Glass Microbarcodes,” Proceedings of the National Academy of Sciences 100, no. 2 (2003): 389–393, 10.1073/pnas.0236044100. PMC141004 12515864

[10] D. C. Pregibon , M. Toner , and P. S. Doyle , “Multifunctional Encoded Particles for High‐throughput Biomolecule Analysis,” Science 315, no. 5817 (2007): 1393–1396, 10.1126/science.1134929. 17347435

[11] H. Lee , J. Kim , H. Kim , J. Kim , and S. Kwon , “Colour‐barcoded Magnetic Microparticles for Multiplexed Bioassays,” Nature Materials 9, no. 9 (2010): 745–749, 10.1038/nmat2815. 20729849

[12] K. W. Bong , S. C. Chapin , and P. S. Doyle , “Magnetic Barcoded Hydrogel Microparticles for Multiplexed Detection,” Langmuir 26, no. 11 (2010): 8008–8014, 10.1021/la904903g. 20178351 PMC2877154

[13] D. C. Appleyard , S. C. Chapin , R. L. Srinivas , and P. S. Doyle , “Bar‐coded Hydrogel Microparticles for Protein Detection: Synthesis, Assay and Scanning,” Nature Protocols 6, no. 11 (2011): 1761–1774, 10.1038/nprot.2011.400. 22015846

[14] F. Zhang , R. C. Haushalter , R. W. Haushalter , et al., “Rare‐Earth Upconverting Nanobarcodes for Multiplexed Biological Detection,” Small 7, no. 14 (2011): 1972–1976, 10.1002/smll.201100629. 21726042 PMC3516929

[15] Y. Zhao , Z. Xie , H. Gu , et al., “Multifunctional Photonic Crystal Barcodes from Microfluidics,” NPG Asia Materials 4, no. 9 (2012): 25, 10.1038/am.2012.46.

[16] S. Han , H. J. Bae , J. Kim , et al., “Lithographically Encoded Polymer Microtaggant Using High‐Capacity and Error‐Correctable QR Code for Anti‐Counterfeiting of Drugs,” Advanced Materials 24, no. 44 (2012): 5924–5929, 10.1002/adma.201201486. 22930454

[17] J. Lee , P. W. Bisso , R. L. Srinivas , J. J. Kim , A. J. Swiston , and P. S. Doyle , “Universal Process‐inert Encoding Architecture for Polymer Microparticles,” Nature Materials 13, no. 5 (2014): 524–529, 10.1038/nmat3938. 24728464

[18] L. N. Kim , M. Kim , K. Jung , et al., “Shape‐encoded Silica Microparticles for Multiplexed Bioassays,” Chemical Communications 51, no. 60 (2015): 12130–12133, 10.1039/c5cc02048d. 26125980

[19] B. Mir‐Simon , I. Reche‐Perez , L. Guerrini , N. Pazos‐Perez , and R. A. Alvarez‐Puebla , “Universal One‐pot and Scalable Synthesis of SERS Encoded Nanoparticles,” Chemistry of Materials 27, no. 3 (2015): 950–958, 10.1021/cm504251h.

[20] H. Q. Nguyen , B. C. Baxter , K. Brower , et al., “Programmable Microfluidic Synthesis of over One Thousand Uniquely Identifiable Spectral Codes,” Advanced Optical Materials 5, no. 3 (2017): 1600548, 10.1002/adom.201600548. 28936383 PMC5604317

[21] Y. Feng , A. K. White , J. B. Hein , E. A. Appel , and P. M. Fordyce , “MRBLES 2.0: High‐throughput Generation of Chemically Functionalized Spectrally and Magnetically Encoded Hydrogel Beads Using a Simple Single‐layer Microfluidic Device,” Microsystems & Nanoengineering 6, no. 1 (2020): 109, 10.1038/s41378-020-00220-3. 33299601 PMC7704393

[22] A. R. Anwar , M. Mur , and M. Humar , “Microcavity‐ and Microlaser‐based Optical Barcoding: A Review of Encoding Techniques and Applications,” ACS Photonics 10, no. 5 (2023): 1202–1224, 10.1021/acsphotonics.2c01611. 37215324 PMC10197175

[23] F. Hu , C. Zeng , R. Long , et al., “Supermultiplexed Optical Imaging and Barcoding With Engineered Polyynes,” Nature Methods 15, no. 3 (2018): 194–200, 10.1038/nmeth.4578. 29334378 PMC5831481

[24] F. Kawasaki , T. Mimori , Y. Mori , et al., “Computational Design of Synthetic Optical Barcodes in Microdroplets,” Advanced Optical Materials 12, no. 12 (2024): 2302564, 10.1002/adom.202302564.

[25] N. Martino , H. Yan , G. Abbott , et al., “Large‐scale Combinatorial Optical Barcoding of Cells With Laser Particles,” Light: Science & Applications 14 (2025): 148, 10.1038/s41377-025-01809-x. PMC11962087 40169572

[26] D. Dendukuri , D. C. Pregibon , J. Collins , T. A. Hatton , and P. S. Doyle , “Continuous‐flow Lithography for High‐throughput Microparticle Synthesis,” Nature Materials 5, no. 5 (2006): 365–369, 10.1038/nmat1617. 16604080

[27] K. W. Bong , K. T. Bong , D. C. Pregibon , and P. S. Doyle , “Hydrodynamic Focusing Lithography,” Angewandte Chemie International Edition 49, no. 1 (2010): 87–90, 10.1002/anie.200905229. 19950160

[28] K. W. Bong , J. Xu , J. H. Kim , et al., “Non‐polydimethylsiloxane Devices for Oxygen‐free Flow Lithography,” Nature Communications 3, no. 1 (2012): 805, 10.1038/ncomms1800. 22549834

[29] M. A. Sahin , H. Werner , S. Udani , D. Di Carlo , and G. Destgeer , “Flow Lithography for Structured Microparticles: Fundamentals, Methods and Applications,” Lab on a Chip 22, no. 21 (2022): 4007–4042, 10.1039/d2lc00421f. 35920614

[30] P. Panda , S. Ali , E. Lo , et al., “Stop‐flow Lithography to Generate Cell‐laden Microgel Particles,” Lab on a Chip 8, no. 7 (2008): 1056–1061, 10.1039/b804234a. 18584079 PMC2790079

[31] E. Stellwagen and N. C. Stellwagen , “Determining the Electrophoretic Mobility and Translational Diffusion Coefficients of DNA Molecules in Free Solution,” Electrophoresis 23 (2002): 2794–2803, 10.1002/1522-2683(200208)23:16<2794::AID-ELPS2794>3.0.CO;2-Y. 12210184

[32] C. Stringer , T. Wang , M. Michaelos , and M. Pachitariu , “Cellpose: A Generalist Algorithm for Cellular Segmentation,” Nature Methods 18, no. 1 (2021): 100–106, 10.1038/s41592-020-01018-x. 33318659

