# Supplementary material for: Decoupling Fabrication From Encoding: DNA‐Addressable Template Microparticles for Large, User‐Defined Optical Barcode Libraries
Source: Adv Sci (Weinh). 2026 Sep 14:e77407. Online ahead of print. doi: 10.1002/advs.77407 (PMC13574990; doi:10.1002/advs.77407)
Supplement: Supplementary file 1 [file ADVS-9999-e77407-s001.docx]

***Supporting Information***

Decoupling Fabrication from Encoding: DNA-Addressable Template Microparticles for Large, User-Defined Optical Barcode Libraries

Akihiro Eguchi,^†[a]^ Yuichiro Iwamoto,^†[a]^ Hinata Tokuda,^†[a]^ Haruka Narita,^†[a]^ Adrian M. Martin,^[a]^ and Sadao Ota*^[a]^

*^[a]^Research Center for Advanced Science and Technology, The University of Tokyo, 4-6-1, Komaba, Meguro-ku, Tokyo 153-8904, Japan*

*^†^These authors contributed equally to this work.*

**email:* *sadaota@g.ecc.u-tokyo.ac.jp*

**Figure S1.** Structural design of the two-PDMS-layer bonded microfluidic device. The device integrates five parallel reactive laminar streams with three-dimensional sheath flows surrounding the reactive core from the top, bottom, and lateral side walls. This configuration enables contact-free transport of the photocurable precursor stream through the UV exposure region, thereby reducing wall fouling during continuous high-throughput particle generation.

**Figure S2.** Quantitative characterization of the quality and reproducibility of five-domain template microparticles. (A) Quantification of domain width in template microparticles fabricated. Particles were stained in an alternating RGBRG pattern to visualize all five DNA-addressable domains. Domain width was determined as the full width at half maximum (FWHM) of the fluorescence intensity profile for each domain. Violin plots show the distribution of domain widths for each fabrication batch (n = 7,720, 7,515, and 5,535 for each batch). (B) Fluorescence leakage was quantified as the fitted fluorescence intensity at the center of the adjacent domain, normalized to the fitted peak intensity of the source domain. The white line indicates the median. The fitted fluorescence intensity was obtained from the Gaussian profile of the source domain (n = 7,720, 7,515, and 5,535 for each batch).

**Figure S3.** Programmable post-fabrication encoding by domain-selective hybridization (A) Fluorescence intensity histograms for three different batches of the beads, showing three resolvable non-zero levels per fluorophore obtained by titrating staining concentrations (values indicated), enabling four addressable levels including the unstained state (n = 1,000). (B) Box plots showing the distribution of the fluorescence intensity across the three different batches of the beads. The center line indicates the median, the box indicates the interquartile range (IQR), and the whiskers extend to the most extreme data points within 1.5 × IQR. CVs are calculated based on the median fluorescence intensity of each batch (n = 1,000).

**Figure S4.** Schematic illustration of split-pool-based particle coloring.

**Figure S5.** 2D intensity scatter plot of AF488 and AF647 signals from the domains 1, 3, 4, and 5 of the beads, exhibiting nine distinct clusters. The colors indicate the nine clusters assigned by GMM.

**Figure S6.** Characterization of beads rejected by posterior-confidence filtering. AF488–AF647 intensity scatter plots for each domain with beads failing the posterior threshold of 0.95 highlighted in orange. Rejected beads were enriched near boundaries between neighboring GMM-defined spectral clusters, indicating ambiguous state assignment, rather than being concentrated in low-intensity or low-signal regions.

**Figure S7.** (A) Overlay of fluorescence intensity distributions (± 2σ) for AF488 and AF647 measured on day 0 (blue) and after 11 days (orange). (B) Ratio of the median value of the AF488 and AF647 fluorescence intensity between day 0 and day 11.

**Figure S8.** Heatmap of matching certainty scores before softmax normalization between beads imaged on day 0 and day 11, highlighting 100 beads out of 10,654 beads.

**Supplementary Tables**

**Table S1. Particle numbers at each stage of the fabrication, encoding, imaging, and decoding workflow.** (*) Only part of the well was imaged, and particles intersecting the image boundaries were excluded from Cellpose segmentation; therefore, the difference between 145,000 and 79,342 does not represent particle loss. (**) Correct five-domain morphology and successful domain-specific hybridization were evaluated jointly because five-domain assignment was based on the domain-specific fluorescence pattern and these criteria could not be quantified separately.

**Table S2.** List of the sequence of the oligonucleotides used in Figure 2 and 5.

**Table S3.** List of the sequence of the oligonucleotides used in Figure 3 and 4.

**Table S4.** Staining conditions used for split-pool library generation used in Figure 4.

**Table S5.** Staining conditions used for split-pool library generation used in Figure 5.
